# Zein-Stabilized Nanospheres as Nanocarriers for Boosting the Aphrodisiac Activity of Icariin: Response Surface Optimization and In Vivo Assessment

**DOI:** 10.3390/pharmaceutics14061279

**Published:** 2022-06-16

**Authors:** Hani Z. Asfour, Nabil A. Alhakamy, Usama A. Fahmy, Osama A. A. Ahmed, Waleed Y. Rizg, Raed I. Felimban, Ashraf B. Abdel-Naim, Mohammad A. S. Abourehab, Rasha A. Mansouri, Ulfat M. Omar, Shaimaa M. Badr-Eldin

**Affiliations:** 1 Department of Medical Microbiology and Parasitology, Faculty of Medicine, King Abdulaziz University, Jeddah 21589, Saudi Arabia; hasfour@kau.edu.sa; 2 Department of Pharmaceutics, Faculty of Pharmacy, King Abdulaziz University, Jeddah 21589, Saudi Arabia; nalhakamy@kau.edu.sa (N.A.A.); uahmedkauedu.sa@kau.edu.sa (U.A.F.); oaahmed@kau.edu.sa (O.A.A.A.); wrizq@kau.edu.sa (W.Y.R.); 3 Center of Excellence for Drug Research and Pharmaceutical Industries, King Abdulaziz University, Jeddah 21589, Saudi Arabia; 4 Mohamed Saeed Tamer Chair for Pharmaceutical Industries, King Abdulaziz University, Jeddah 21589, Saudi Arabia; 5 Department of Medical Laboratory Sciences, Faculty of Applied Medical Sciences, King Abdulaziz University, Jeddah 21589, Saudi Arabia; faraed@kau.edu.sa; 6 Center of Innovation in Personalized Medicine (CIPM), 3D Bioprinting Unit, King Abdulaziz University, Jeddah 21589, Saudi Arabia; 7 Department of Pharmacology and Toxicology, Faculty of Pharmacy, King Abdulaziz University, Jeddah 21589, Saudi Arabia; aaabdulrahman1@kau.edu.sa; 8 Department of Pharmaceutics and Industrial Pharmacy, College of Pharmacy, Minia University, Minia 61519, Egypt; maabourehab@uqu.edu.sa; 9 Department of Pharmaceutics, Faculty of Pharmacy, Umm Al-Qura University, Makkah 21955, Saudi Arabia; 10 Department of Biochemistry, Faculty of Sciences, King Abdulaziz University, Jeddah 21589, Saudi Arabia; amansouri@kau.edu.sa (R.A.M.); uomer@kau.edu.sa (U.M.O.); 11 Princess Dr. Najla Bint Saud Al- Saud Center for Excellence Research in Biotechnology, King Abdulaziz University, Jeddah 21589, Saudi Arabia; 12 Department of Pharmaceutics, Faculty of Pharmacy, Cairo University, Cairo 11562, Egypt

**Keywords:** icariin, D-α-Tocopherol polyethylene glycol 1000 succinate, zein, sodium deoxycholate, in vivo assessment, sexual behavior

## Abstract

Icariin (ICA), a main active compound of the Epimedium genus, is used as an aphrodisiac in traditional Chinese herbal medicine. Despite its therapeutic efficacy, ICA displays reduced oral absorption, and therefore, low bioavailability hindered its clinical application. Implementing nanotechnology in the field of formulation has been a focus to improve the efficacy of ICA. In this regard, polymeric nanoparticles find a potential application as drug delivery systems. A nanosphere formula was designed, aiming to improve the drug’s efficacy. The proposed ICA nanosphere formula (tocozeinolate) was optimized using D-optimal response surface design. The concentrations of ICA (X_1_), D-α-tocopherol polyethylene glycol 1000 succinate (TPGS, X_2_), zein (X_3_), and sodium deoxycholate (SDC, X_4_) expressed as percentages were investigated as quantitative independent variables. As per the experimental design, 23 formulations were developed, which were investigated for particle size (PS, nm), zeta potential (ZP, mV), and entrapment efficiency (EE, %) as response parameters. Numerical optimization and desirability approach were employed to predict the optimized variable levels that, upon combination, could result in minimized size and maximized zeta potential and ICA entrapment. The optimized ICA–tocozeinolate nanospheres showed a particle size of 224.45 nm, zeta potential of 0.961 mV, and drug entrapment of 65.29% that coincide well with the predicted values. The optimized ICA–tocozeinolate nanospheres were evaluated for sexual behavior in Wistar male rats compared to raw ICA at equivalent doses (20 mg/kg). In vivo assessment results showed significant sexual behavior enhancement by the optimized formulation, as evidenced by decreased average time of both mount latency (ML) and ejaculation latency (EL) to almost half those of raw ICA. Additionally, intromission latency (IL) time was reduced by 41% compared to the raw ICA. These results highlighted the potential of the proposed ICA–tocozeinolate nanospheres as a promising platform for improving the delivery and efficacy of therapeutic agents.

## 1. Introduction

Icariin (ICA) is a prenylated flavonol glycoside and the main active compound of Herba Epimedii [1]. ICA is used as an aphrodisiac, to improve cardiovascular function, as an antirheumatic, and to combat neurodegenerative disorders in traditional Chinese herbal medicine [2,3,4,5,6]. ICA is a diglycoside that is not readily absorbed [7,8]. Its use in Chinese traditional medicine ICA as a tonic and a potent enhancer of erectile function has been well established for centuries. Epimedium extract (rich in ICA) shows vasodilating effects facilitated by NO release. The extract performs a vasokinetic action on arteries and arterioles [8,9]. ICA is a cGMP-specific phosphodiesterase type 5 inhibitor that represents an effective orally administered supplement for erectile dysfunction management [10,11]. ICA induces neuroprotective effects in vitro and in vivo and can improve dopaminergic neuronal loss and neuroinflammation in mice [12]. Furthermore, ICA reduces brain dysfunction induced by lipopolysaccharide and corticosterone-induced neuron apoptosis [13,14]. ICA inhibited both MAO-A and MAO-B activities and improved experimentally decreased brain monoamine neurotransmitter levels. Therefore, ICA enhanced brain monoamine content, particularly dopamine [13]. Incidentally, decreased male sexual desire has been linked to decreased dopamine levels. This was confirmed by the observation that treatment of erectile dysfunction by the use of dopamine receptor agonist apomorphine offers strong support for the participation of the dopaminergic system in the control of sexual function [15]. Thus, it can be suggested that ICA enhances male libido, at least partly, via boosting dopamine systems in different brain areas.

Despite ICA’s wide range of therapeutic efficacy, its reduced oral absorption and hence low bioavailability hindered its clinical application [8,16]. The scientific community has focused its research effort in this area on novel formulae to improve the efficacy of ICA. In this regard, polymeric nanoparticles find a major application as a drug delivery system owing to their promising advantages [17].

Utilizing naturally derived polymers for the nanoformulation of therapeutic agents has gained increased interest [18]. Zein nanoparticles are promising candidates for controlling the release of hydrophobic active pharmaceuticals as a result of hydrophobicity and biodegradable zein characters [11]. Sodium deoxycholate (SDC) is a bile salt utilized in formulation research, owing to its membrane-destabilizing activity, for penetration enhancement [19,20].

D-α-Tocopherol polyethylene glycol 1000 succinate (TPGS), the water-soluble form of vitamin E, is the ester form of vitamin E and a PEG chain. Because of its unique amphiphilic structure, it has excellent drug delivery capability [21,22,23]. In drug delivery, TPGS is formulated with various types of drugs, particularly those classified as biopharmaceutics classification system (BCS) classes II and IV. Furthermore, it has been reported that TPGS improved intestinal lymphatic transport [24,25]. 

Accordingly, this study aimed at developing a novel nanocarrier that could combine the advantages of zein, TPGS, and SDC as well as the nanosize of the formulation (tocozeinolate) to enhance the sexual behavior of ICA. D-optimal design was implemented for response surface optimization of the proposed formulation to obtain minimized size, maximized zeta potential and entrapment efficiency. The optimized formulation was subjected to in vitro characterization and in vivo assessment of sexual behavior in Wistar rats.

## 2. Materials and Method

### 2.1. Materials

Icariin, zein, TPGS, and SDC were purchased from Sigma-Aldrich, Burlington, MA, USA. All solvents and chemicals were of analytical grade.

### 2.2. Preparation of ICA–Tocozeinolate Nanospheres

The preparation of ICA–tocozeinolate nanospheres was carried out by dissolving specified amounts of ICA and TPGS (according to the experimental design) in pure ethanol (25 mL). Amounts of zein specified by the design were dissolved in 90% ethanol. Both alcoholic solutions were mixed by stirring. SDC (specified amounts) was dissolved in distilled water. The aqueous solution was then mixed with the alcoholic solution by stirring. The prepared solution was kept stirred at 400 rpm for 30 min using a stirring hotplate. After that, the solution was subjected to rotary evaporation (R200, Büchi Labortechnik AG, Flawil, Switzerland) at 35 °C until complete evaporation of the alcoholic part was achieved. The prepared solution was centrifuged at 20,000 rpm (Sigma 3k30, Osterode, Germany) for 45 min and washed with double-distilled water; the centrifugation and washing cycle was repeated twice, then the sample was subjected to lyophilization. 

### 2.3. Experimental Design and Optimization of ICA–Tocozeinolate Nanospheres

The proposed ICA–tocozeinolate nanosphere formula was optimized using D-optimal response surface experimental design. The concentrations of ICA (X_1_), TPGS (X_2_), Zein (X_3_), and SDC (X_4_)—expressed as percentages—were investigated as quantitative independent variables. The levels of the four variables are indicated in Table 1. Particle size (PS, nm, Y_1_), zeta potential (ZP, mV, Y_2_), and entrapment efficiency (EE, %, Y_3_) were selected as response parameters. As per the selected design, 23 experimental runs, including three lack-of-fit points, four replicate points, and an additional center point were generated by Design-Expert software (Version 12; Stat-Ease Inc., Minneapolis, MN, USA); the combinations of variables’ levels for each experimental run are listed in Table 2. The optimal model fitting the data of each response was selected from linear, two-factor interaction (2FI), and quadratic models based on the computed, predicted, and adjusted determination coefficients (R^2^) as well as the predicted residual sum of squares (PRESS). The goodness of data fitting was assessed using the diagnostic plots generated by the software. The terms’ coefficients in the equations expressing the best-fitting model for each response were utilized to predict the relative magnitude of the corresponding variable or interaction’s impact. Analysis of variance (ANOVA) was utilized to assess the studied variables’ significance and their interaction at *p* < 0.05. The investigated variables’ effects and their interactions were graphically illustrated using two-dimensional contour and three-dimensional response surface plots. Numerical optimization and desirability approaches were applied to the measured data to predict the optimal variable levels to achieve the desired set goals for the responses upon combination, Table 1.

### 2.4. In Vitro Characterization 

#### 2.4.1. Particle Size and Zeta Potential

ICA–tocozeinolate evaluation was carried out by water (double-distilled) dilution using a Zetasizer Nano ZSP particle size analyzer instrument (Malvern, UK). 

#### 2.4.2. Entrapment Efficiency (EE%)

Entrapment efficiency (EE%) of ICA within the ICA–tocozeinolate nanospheres was determined by indirect method determination [26]. Briefly, 1 mL of the formulated ICA–tocozeinolate was added to 4 mL of deionized water, and the mixture was centrifuged at 15,000 rpm for 15 min. One milliliter of the clear supernatant was mixed with 4 mL methanol., Next, 100 uL of the vortexed solution and 2 mL of acetonitrile were mixed, then thoroughly stirred for 1 min and centrifuged at 5000 rpm for 20 min. The supernatant was subsequently evaporated in a clean glass tube. The residue was then reconstituted with 200 uL of mobile phase (0.1% formic acid (A) and acetonitrile (B) gradient system) and analyzed by high-performance liquid chromatography (HPLC) utilizing Agilent 1260 (Agilent Technologies, Santa Clara, CA, USA) with a diode-array detector and analyzed at 270 nm [27]. 

#### 2.4.3. Fourier-Transform Infrared Spectroscopy (FTIR) 

FTIR spectra of the optimized ICA–tocozeinolate nanosphere formula and single formula components (ICA, SDC, TPGS, and zein) were investigated at 4000–400 cm^−1^ using a Tensor 37, FTIR spectrometer (Bruker, Fremont, CA, USA).

#### 2.4.4. X-ray Diffraction (XRD)

The XRD pattern of the optimized ICA–tocozeinolate formulation was investigated utilizing a XMD-300 X-ray diffractometer (Unisantis Europe GmbH, Osnabrück, Germany). Intensities were measured at 2θ intervals of 0.02°.

#### 2.4.5. Transmission Electron Microscope (TEM)

The shape of optimized ICA–tocozeinolate nanospheres was explored using a JEOL GEM-1010 (JEOL Ltd., Akishima, Tokyo, Japan) transmission electron microscope (TEM) at 80 kV at The Regional Center for Mycology and Biotechnology, (RCMB) Al-Azhar University, Cairo, Egypt. The sample was suspended in distilled water and one drop of the sample was spread on a carbon-coated grid, then allowed to be dried at ambient temperature. In addition, 1% phosphotungstic acid was used for negative staining of the sample. Then, the sample was dried at ambient temperature for 15 min before visualization. 

### 2.5. Optimized ICA- Tocozeinolate Release 

Using the cellulose tube diffusion method, in vitro release of ICA from tocozeinolate was performed [26]. The cellulose tube was soaked in release media overnight. The cellulose tube was loaded with 2 mL ICA–tocozeinolate aqueous dispersion (equivalent to 10 mg ICA). After being securely sealed, it was placed in a receptor compartment holding 500 mL of phosphate buffer (pH 7.4). The ICA was released at 37 ± 0.5 °C utilizing the U.S. Pharmacopoeia dissolution device (paddle method) at 100 rpm. At specified time intervals of 0.5, 1.0, 2.0, 4.0, 6.0, 8.0, 12.0, and 24.0 h, two milliliters of the released media was removed from the dissolving vessels and replaced with an equal volume of fresh media at the same temperature. Prior to HPLC analysis (as indicated in EE% method), all of the collected released samples were filtered using a 0.45 μm syringe filter. This analysis was conducted in triplicate.

### 2.6. Acute Toxicity Investigation Optimized ICA–Tocozeinolate Formulation

The oral administration of a single dose of optimized ICA–tocozeinolate formulation (2000 mg/kg) to three experimental female rats was carried out to investigate mortality according to the rules outlined by the Organization for Economic Co-operation and Development (OECD). The same process was performed with three additional rats after 24 h, and the outcome was investigated (guideline No. 423, 2002).

### 2.7. In Vivo Assessment of the Effect of Optimized ICA–Tocozeinolate Nanospheres on Male Rat Sexual Behavior

Wistar rats (210–240 g) of both sexes were obtained from the animal facility of King Abdulaziz University (KAU) and housed in a 12 h light–dark cycle and a temperature of 22 ± 2 °C. The animal care procedures were certified by the Faculty of Pharmacy’s Research Ethics Committee (PH-1443-27). Twenty-four male rats were divided into 4 groups of 6 rats each. The control group received 3 mL/kg of 0.5 carboxymethyl cellulose (CMC), whilst the other 3 groups received vehicle—raw ICA suspended in CMC (50 mg/kg)—and ICA–tocozeinolate (equivalent to 50 mg/kg ICA) as a single oral daily dose for 10 days. Male and female rats were mated, and sexual behavior parameters were evaluated in the first period of the dark cycle of day 11. The sexual behavior of the males was observed by well-trained technicians without prior knowledge of the study details. Observations were achieved in an air-conditioned, sound-attenuated room lit with a faint red light. Single male rats were transferred into rectangular glass monitoring cages (40 × 50 × 40 cm) and allowed to become accustomed to the testing chamber for 15 min. Then, female rats were introduced into the cages (1 female per cage). Parameters of sexual behavior were assessed as previously described [10,28]. Mount latency (ML) is defined as time (in seconds) from the introduction of the female to the first mount; ejaculation latency (EL) is defined as time (in seconds) from the first intromission to ejaculation, and intromission latency (IL) is defined as time (in seconds) from introduction of the female to the first intromission (vaginal penetration).

### 2.8. Statistical Analysis

Data are presented as mean ± SD. Statistical analysis was performed using one-way ANOVA followed by Tukey’s post hoc test for multiple comparisons. A level of probability of 0.05 was used as the criterion for significance. All statistical analyses were performed using GraphPad Prism software version 8.1 (La Jolla, CA, USA).

## 3. Results 

### 3.1. D-Optimal Response Surface Design

#### 3.1.1. Fit Statistics and Diagnostic Analysis

Fit statistical analysis results for the responses, namely, size, zeta potential, and entrapment efficiency are presented in Table 3. On the basis of the highest R^2^ and lowest PRESS, the vesicle size data fitted the 2FI model; the zeta potential fitted the linear model, while the entrapment efficiency fitted the quadratic model. The adjusted R^2^ and the predicted R^2^ for each response exhibited appropriate coincidence with difference of less than the permissible limit of 0.2, verifying the model’s validity. Moreover, the selected models for each response exhibited adequate precision values greater than the desirable value of 4, indicating an appropriate signal to noise ratio. According to the previously computed parameters, the selected models could be adequately utilized to explore the experimental design space.

**Figure 1:**
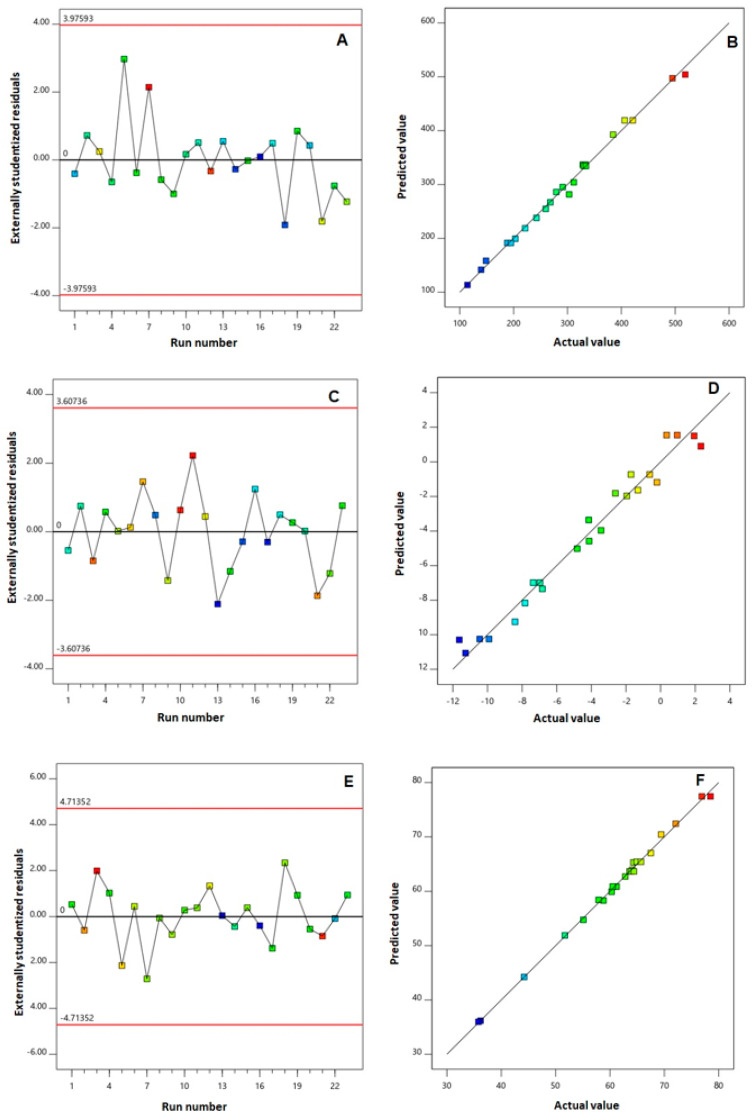
Diagnostic plots for ICA–tocozeinolate nanosphere size (**A**,**B**), zeta potential (**C**,**D**), and EE% (**E**,**F**) for the measured responses of ICA–tocozeinolate nanospheres. Externally studentized residuals vs. run number plot (**A**,**C**,**E**) and (**B**,**D**,**F**) normal probability plot.

The goodness of fit of the selected models was further verified via developing diagnostic charts, shown in Figure 1. The colored points in the externally studentized residuals vs. run plots, Figure 1A,C,E, were scattered randomly within the limits (illustrated by the red lines), indicating the absence of any lurking variable that could exert an influence on any of the measured responses. Moreover, the predicted versus actual plots, illustrated in Figure 1B,D,F, showed highly linear patterns, revealing that the observed responses showed good analogy to the predicted ones [22,29].

#### 3.1.2. Influence of Variables on Particle Size (Y_1_)

ANOVA for size indicated the 2FI model’s significance, as evidenced by the F-value of 225.57 (*p* < 0.0001). The lack-of-fit F-value of 3.64 (*p* = 0.1138) shows a non-significant lack of fit; thus, fitting of the measured size to the recommended model is ensured. The equation (Equation (1)) showing the 2FI model in terms of coded factor was generated by the software.

**Y_1_
*(particle size)*** = 286.02 + 40.15 X_1_ − 64.99 X_2_ − 45.04 X_3_ − 80.35 X_4_ + 1.61 X_1_X_2_ − 2.69 X_1_X_3_ − 4.20 X_1_X_4_ + 4.82 X_2_X_3_ + 10.38 X_2_X_4_ + 12.70 X_3_X_4_ (1)

**Figure 2:**
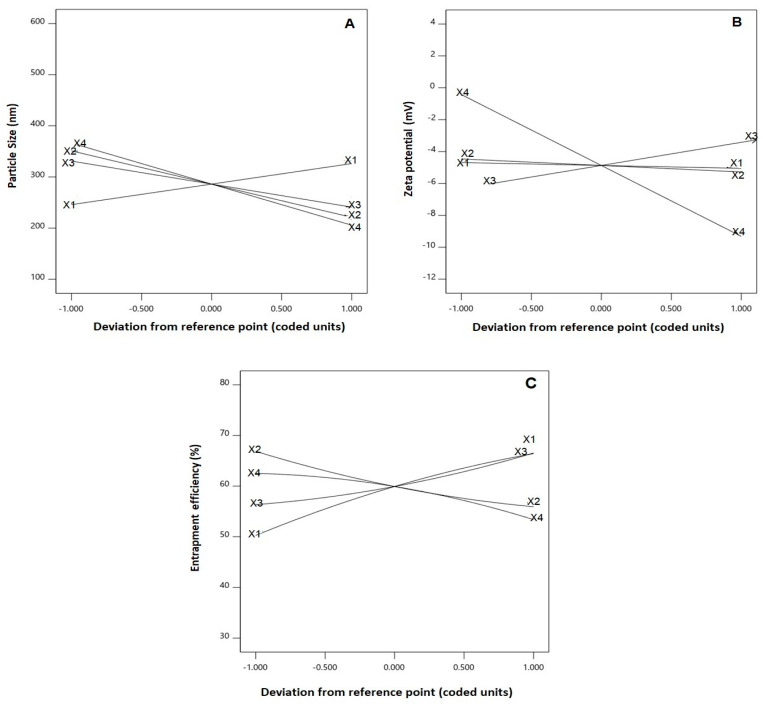
Perturbation graph for the main effects of critical attributes; ICA concentration (X_1_), TPGS concentration (X_2_), zein concentration (X_3_), and SDC concentration (X_4_) on (**A**) particle size, (**B**) zeta potential, and (**C**) EE% of ICA–tocozeinolate nanospheres.

**Figure 3:**
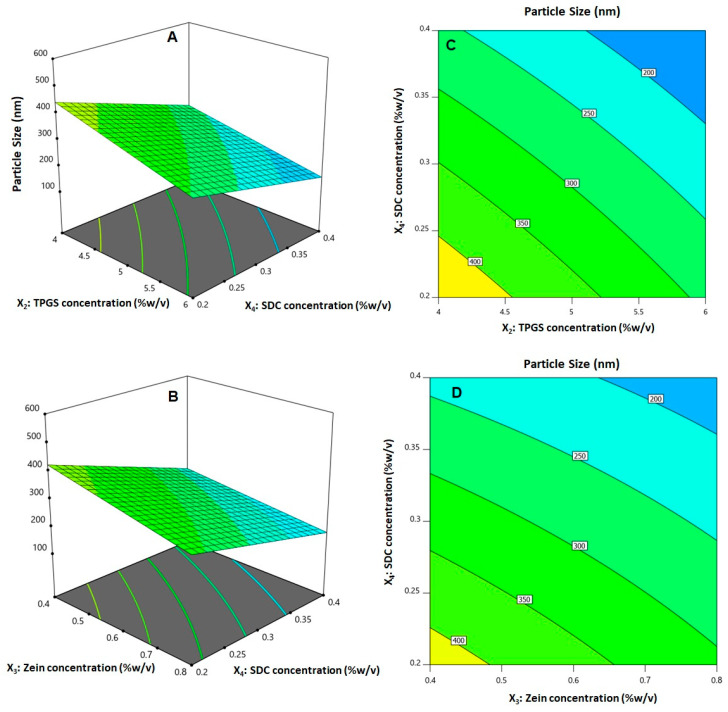
Response surface (**A**,**B**) and contour plots (**C**,**D**) indicating interaction between the significant factors on ICA–tocozeinolate nanosphere size.

The analysis indicated that all the linear terms corresponding to the four investigated variables had a significant effect on size (*p* < 0.0001 for all terms). The interaction terms X_2_X_4_ and X_3_X_4_, representing the interaction between SDC concentration and either TPGS or zein concentrations, respectively, were also significant at *p* < 0.05. Figure 2A illustrates the perturbation graph demonstrating the impact of the studied factors on size, while Figure 3 illustrates the 3D response and the 2D contour plots that demonstrate the interaction between the significant variables. The illustrations show that the nanospheres’ size significantly increases with increasing ICA concentration while decreasing with increasing TPGS, zein, and SDC concentrations. This finding is supported by the positive sign of the X_1_ coefficient and the negative sign of the X_2_, X_3_, and X_4_ coefficients. The order of significance was SDC > TPGS > zein > ICA, as evidenced by the values of the linear terms’ coefficients in the developed Equation (1). 

#### 3.1.3. Influence of Variables on Zeta Potential (Y_2_)

The prepared ICA–tocozeinolate nanospheres exhibited zeta potential values ranging from −11.63 ± 3.4 to 2.33 ± 2.4 mV. It is documented that positively charged nanoparticles could have better permeation ability and accumulation within cancerous tissues and tumor vasculature in comparison to the surrounding environment [30,31]. Therefore, the prepared nanospheres were optimized with the aim of maximizing the value of the zeta potential. ANOVA for zeta potential indicated the significance of the linear model as depicted by the corresponding F-value of 163.92 (*p* < 0.0001), respectively. The lack-of-fit F-value of 2.88 (*p* = 0.1582) reflects non-significant lack of fit; thus, the fitting of zeta potential values to the proposed model is confirmed. Equation (2) shows the linear model for the zeta potential in coded factor terms.

**Y_2_
*(zeta potential)*** = − 4.58 − 0.1813 X_1_ − 0.4082 X_2_ + 1.45 X_3_ − 4.45 X_4_ (2)

The analysis indicated that the linear terms X_2_, X_3_, and X_4_—corresponding to concentration of TPGS, zein, and SDC, respectively—revealed significance on zeta potential (*p* = 0.041 for X_2_ and *p* < 0.0001 for X_3_ and X_4_). The extremely lower *p*-values of the coefficients X_3_ and X_4_ reveal that zein and SDC concentrations play an important role in the zeta potential value. Further, SDC concentration possess the highest impact, as proved by the highest coefficient for the X_4_ term. Figure 2B shows the main effects of the studied factors on zeta potential. The illustration shows that the zeta potential significantly increases at higher zein concentration and lower TPGS and SDC concentrations. The positive sign of X_3_ and the negative signs of the X_2_ and X_4_ coefficients support this observation. The effect of SDC concentration was the most prominent effect on zeta potential, as depicted by the highest corresponding linear term coefficient in Equation (2). 

#### 3.1.4. Influence of Variables on Entrapment Efficiency (Y_3_)

The prepared ICA–tocozeinolate nanospheres exhibited wide variation in entrapment efficiency, ranging from 44.2 ± 7.1 to 78.5 ± 4.3 %. Aiming to maximize ICA entrapment, the influence of formulation factors on entrapment efficiency was studied. ANOVA for entrapment efficiency provided further proof for the quadratic model significance, as evidenced by the F-value of 247.38 (*p* < 0.0001). The lack-of-fit F-value of 2.11 (*p* = 0.2436) shows a non-significant lack of fit; thus, assuring the fitting of the entrapment efficiency data to the recommended model was accomplished. Equation (3), which reveals the coded factor terms of the quadratic model, was generated by the software.

**Y_3_
*(entrapment efficiency)*** = 59.92 + 8.08 X_1_ − 5.47 X_2_ + 5.01 X_3_ − 4.56 X_4_ + 2.02 X_1_X_2_ − 1.94 X_1_X_3_ + 1.66 X_1_X_4_ − 0.89 X_2_X_3_ − 0.047 X_2_X_4_ + 0.34 X_3_X_4_ − 1.59 X_1_^2^ + 1.48 X_2_^2^ + 1.52 X_3_^2^ − 1.98 X_4_^2^ (3)

**Figure 4:**
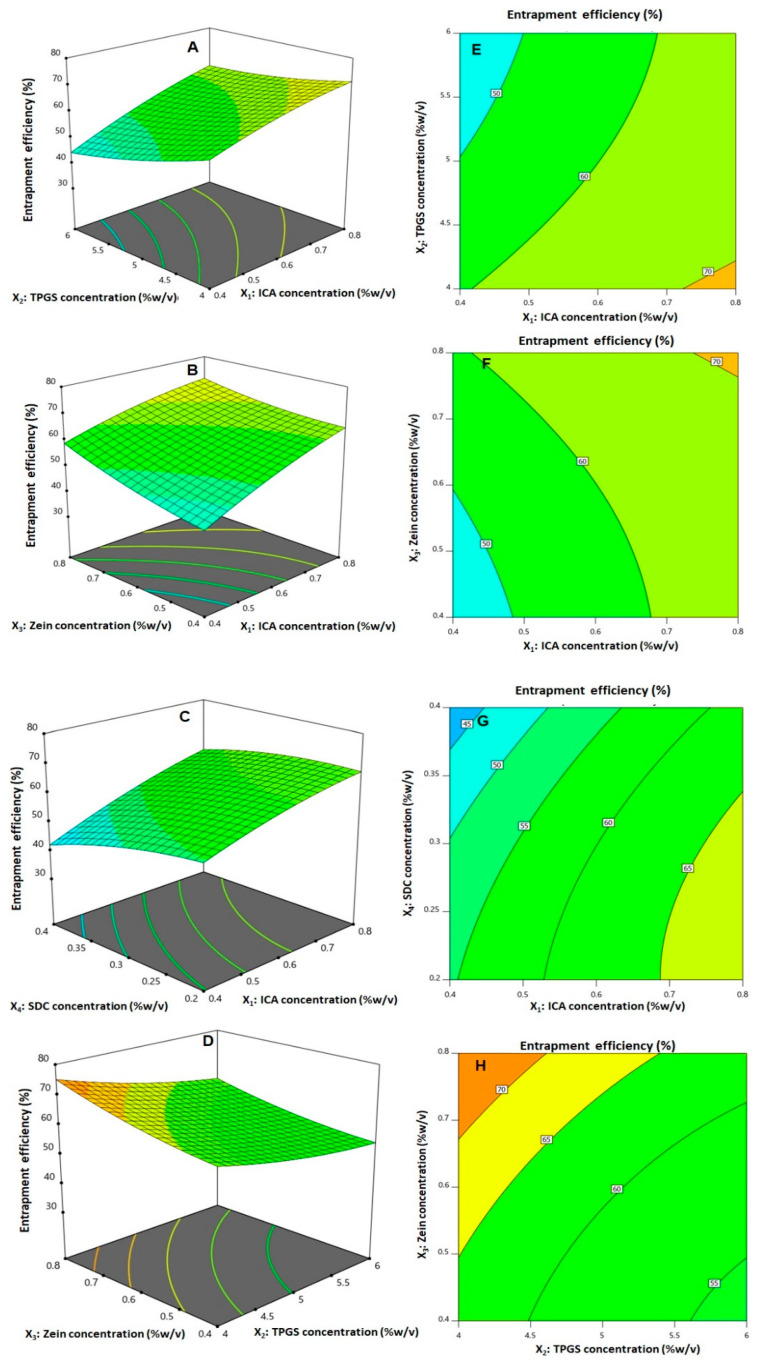
Response surface (**A**–**D**) and contour plots (**E**–**H**) displaying the interaction between the significant factors on the entrapment efficiency of ICA–tocozeinolate nanospheres.

ANOVA results revealed that all the linear terms and the quadratic terms corresponding to the four investigated variables exhibited a markedly significant impact on entrapment efficiency (*p* < 0.0001 for all linear terms; *p* = 0.0192, 0.0160, 0.0314, and 0.0053 for X_1_^2^, X_2_^2^, X_3_^2^, and X_4_^2^, respectively). The interaction terms X_1_X_2_, X_1_X_3_, X_1_X_4_, and X_2_X_3_—representing the interaction between ICA concentration and either TPGS, zein, or SDC concentration and the interaction between TPGS and zein concentrations, respectively—were also found to be significant at *p* < 0.05. Figure 2C illustrates the perturbation graph demonstrating the impact of the studied factors on entrapment, while Figure 4 illustrates the 3D response and the 2D contour plots that represent the interaction between the significant variables. The illustrations show that ICA entrapment significantly increases with increasing ICA and zein concentrations, while it decreases with increasing TPGS and SDC concentrations. This finding is supported by the positive sign of the X_1_ and X_3_ coefficients and the negative sign of the X_2_ and X_4_ coefficients. 

#### 3.1.5. Optimization of ICA–Tocozeinolate Nanospheres

**Figure 5:**
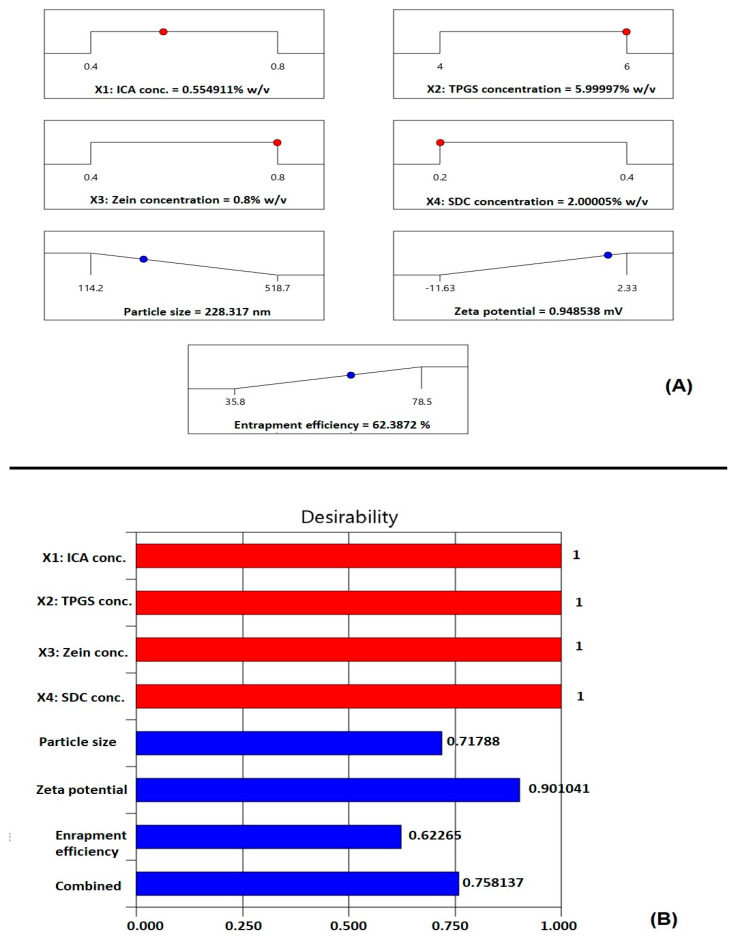
(**A**) Ramp graphs representing the optimized factor levels and the predicted responses for the optimized ICA–tocozeinolate nanospheres. (**B**) Desirability values for the predicted responses and overall desirability of the optimized ICA–tocozeinolate nanospheres.

Numerical optimization and the desirability approach were implemented to predict the optimized variable levels that, upon combination, could result in minimized size and maximized zeta potential and ICA entrapment. The ramp graphs presented in Figure 5A show the optimized levels and the predicted responses, while the desirability for each response and the overall desirability are graphically illustrated in Figure 5B. The optimized formulation composition that could achieve the goals of the optimization process were predicted as follows: ICA concentration (0.55% *w*/*v*), TPGS concentration (5.99% *w*/*v*), zein concentration (0.8% *w*/*v*), and SDC concentration (2.00% *w*/*v*). The measured particle size of 224.45 nm with a polydispersity index of 0.34, zeta potential of 0.961 mV, and drug entrapment of 65.29% coincides well with the predicted values, showing relative percentage errors of 1.69%, 1.26%, and 4.66%, respectively. The relatively small computed percentage errors prove the reliability of the optimization process.

### 3.2. Characterization of Optimized ICA–Tocozeinolate

#### 3.2.1. FTIR

**Figure 6:**
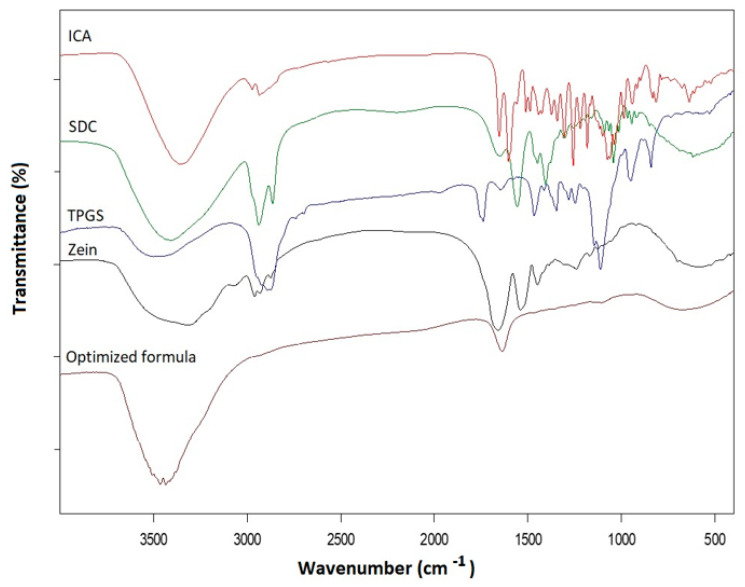
FTIR spectra of the optimized ICA-tocozeinolate nanosphere formula, and single formula components: ICA, SDC, TPGS, and zein.

The FTIR results presented in Figure 6 showed that ICA has a broad absorption band at ≈3350 cm^−1^ related to the hydroxyl groups. ICA also revealed an absorption band (characteristic) at 3000–2800 cm^−1^ related to C–H aliphatic stretching and a peak at ≈1650 cm^−1^ for C=O stretching. SDC FTIR spectrum revealed a broad absorption peak at 3350 cm^−1^ corresponding to the hydroxyl group and a characteristic absorption band before 3000 cm^−1^ corresponding to aliphatic C–H stretching [32]. A C=O ester stretching band at 1735 cm^−1^ was confirmed by an ester C–O stretching band at 1245 cm^−1^. Aditionally, a carbonyl ester stretching peak was revealed at 1690 cm^−1^. Zein spectrum revealed a broad band at 3500–3200 cm^−1^ analogous to the O-H and stretching N-H group confirmed by N-H bending at 1580 cm^−1^. Zein also showed a broad band around 1650 cm^−1^ for multiple amidic bonds of amino acids. TPGS revealed an absorption characteristic band before 3000 cm^−1^ related to aliphatic C–H stretching and a C=O ester stretching band at 1735 cm^−1^, which was confirmed by an ester C–O stretching band at 1245 cm^−1^. In addition, a C=O stretching band at 1738 cm^−1^ in the fatty acid ester and an ester C–O stretching band at 1245 cm^−1^ were revealed. The optimized ICA–tocozeinolate nanosphere spectrum revealed abolishment of the most common feature of all formula components: stretching aliphatic CH_2_ before 3000 cm^−1^ that may be attributed to involvement in non-polar attraction forces (e.g., van der Waals attraction). An increase in both intensity and broadness of the hydroxyl group band occurred at ≈3350 cm^−1^, which is attributed to the intermolecular hydrogen bonding between various formula components. The reduction in intensity and broadness of the C=O stretching band at ≈1700 cm^−1^ could lead to the possibility of either C=O group participation in hydrogen bonding as a hydrogen bond acceptor or be as a result of bulkiness and steric hinderance resulted from the formation of the nanospheres.

#### 3.2.2. XRD

**Figure 7:**
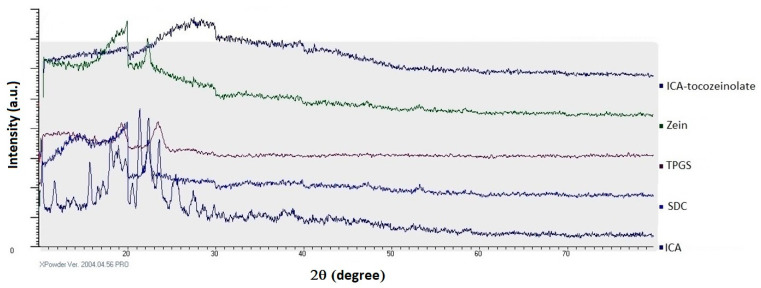
XRD diffraction analysis of the optimized ICA–tocozeinolate nanosphere formula and single formula components: ICA, SDC, TPGS, and zein.

To investigate the physical state of ICA in the optimized ICA–tocozeinolate nanospheres, XRD was carried out. The results in Figure 7 revealed numerous sharp and distinct peaks between 2° and 30°, indicating ICA’s crystalline nature. ICA loading into tocozeinolate nanospheres (optimized formula) revealed the absence of the crystalline peaks of the drug that indicate the transformation of ICA’s crystalline nature into an amorphous form (Figure 7). 

#### 3.2.3. TEM Imaging

**Figure 8:**
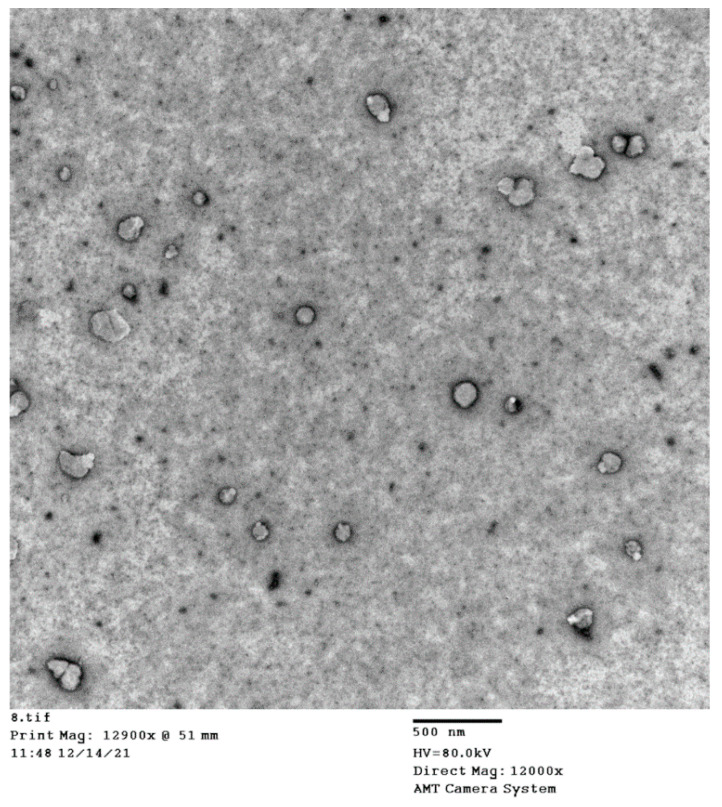
TEM images of the optimized ICA–tocozeinolate nanosphere formula.

The TEM image (Figure 8) of the optimized ICA–tocozeinolate formula revealed almost-spherical nanostructures with a smaller average size than the size obtained by the particle size analyzer. The variation in particles could be related to the drying process during handling the sample for TEM investigation. 

### 3.3. Optimized ICA–Tocozeinolate Release

**Figure 9:**
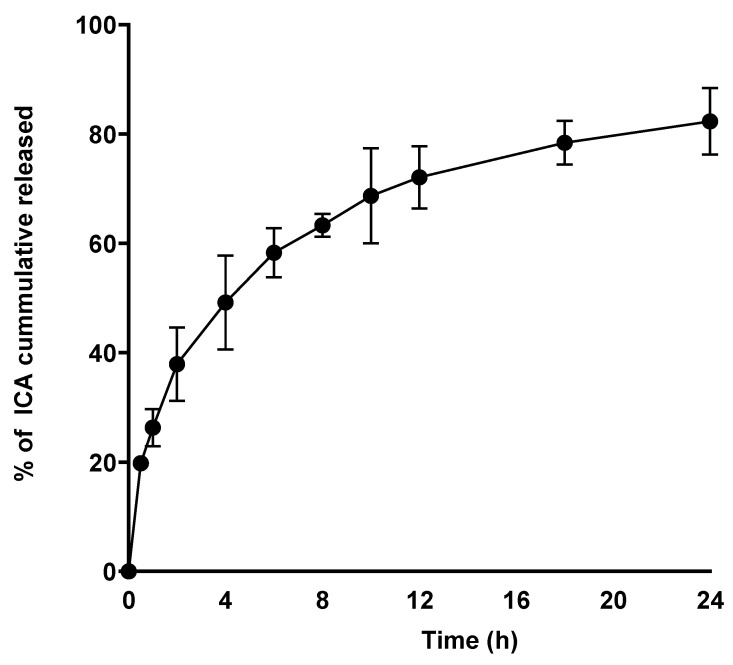
In vitro release profile of ICA from optimized ICA–tocozeinolate.

Figure 9 depicts the in vitro release profile of the optimized ICA–tocozeinolate. It is obvious from the figure that the ICA released demonstrated an initial burst release followed by a slower release pattern; 82.3± 6.1% was reached within 24 h.

### 3.4. Acute Toxicity Investigation Optimized ICA–Tocozeinolate Formulation

The acute toxic class method stated in OECD standards No. 423 indicated that this formulation is Category 5 with an LD50 of nearly 2000 mg/kg (Globally Harmonized System of Classification and Labeling of Chemicals). As a result, the optimized ICA–tocozeinolate formulation is considered nontoxic.

### 3.5. In Vivo Assessment for the Effect of Optimized ICA–Tocozeinolate Nanospheres on Male Rat Sexual Behavior

**Figure 10:**
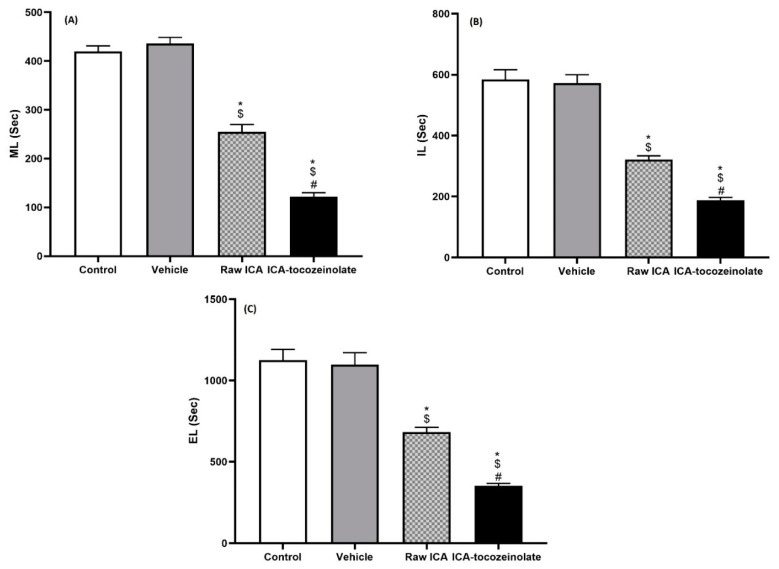
Effect of optimized ICA–tocozeinolate on (**A**) ML, (**B**) IL and (**C**) EL. ML = mount latency = time (in seconds) from the introduction of the female to the first mount. IL = intromission latency = time (in seconds) from introduction of the female to the first intromission (vaginal penetration). EL = ejaculation latency = time (in seconds) from the first intromission to ejaculation. * Significant vs control; $ significant vs vehicle treated control; # Significant vs. ICA.

Oral administration of optimized ICA–tocozeinolate nanospheres resulted in male rat sexual behavior enhancement as compared to raw ICA at equivalent doses (20 mg/kg). This was evidenced by a decrease in average time of ML to almost half that of raw ICA (Figure 10A). Additionally, IL time was reduced by 41% when compared to the raw ICA (Figure 10B). In addition, the optimized ICA–tocozeinolate reduced time of EL to almost half that of raw ICA (Figure 10C).

## 4. Discussion

ICA, as the main component (active compound) of Horny Goat (Herba Epimedii), is a commonly used traditional herbal preparation as a tonic and aphrodisiac in Far East Asian countries [33]. The main concern for ICA is its poor aqueous solubility and oral bioavailability of 0.12 [8]. Nanocarriers could offer a promise to overcome the barriers facing ICA bioavailability through entrapment of the ICA within the core of the nanoparticle and reduction of particle size (hence an increase surface area) and could enhance the solubility of ICA [34,35,36,37,38]. TPGS, with its amphipathic character, is widely investigated for its ability to solubilize poorly water-soluble drugs [39,40]. TPGS is a P-glycoprotein inhibitor and is used as an excipient for overcoming multidrug resistance (MDR) and for improving the bioavailability of orally administered drugs [41,42]. TPGS combined with zein have enhanced bioavailability the isoflavone phytochemical daidzin [43]. SDC has shown enhanced oral drug bioavailability [32,44]. The combination of these promising additives into a nanosphere for encapsulation of ICA requires proper selection of the optimum levels of the components in relation particle size, zeta potential, and ICA entrapment. 

Nanoparticulate systems with size less than 400 nm have recently attracted attention in the arena of drug delivery [45,46]. The prepared optimized ICA–tocozeinolate nanospheres exhibited promising size that ranged from 114.2 ± 15.4 to 538.7 ±37.4 nm. It is reported that a nano-sized delivery system could improve tissue penetration and enhance payload activity [47,48]. Accordingly, size was reduced to its possible minimum value to enhance the surface area available for tissue penetration [49]. Thus, the study aimed at optimizing the nanospheres to minimize size. The increase in nanosphere size at higher drug concentrations was reported previously with drug loading in nanocarrier formulation [50]. The direct relationship between ICA concentration and size could be related to the increased entrapment of the lipophilic compound (ICA) in the developed nanospheres [51]. The reduced size observed at higher SDC concentrations might be attributed to the stabilizing effect of SDC on colloidal nanosphere dispersion with consequent aggregation reduction [52].

Zeta potential value is related to the type and magnitude of the surface charge of nanoparticles. The decreased zeta potential at higher SDC concentrations could be explained by the possible binding between the anionic head groups of SDC with the cationic amino acid moieties of zein protein, which would could cause a marked exposure of the anionic residues [9,52]. It is worth mentioning that although the zeta potential of the optimized formulation was 0.961 mV, other factors than zeta potential can contribute to the stability of nanoparticles; for example, steric stabilization can be the main contributor to nanoformulation stability that could arise from polyethylene glycol hydrophilic part of TPGS. This requires further investigation for confirmation.

The influence of formulation factors on entrapment efficiency was studied with the aim to maximize ICA entrapment. The higher retention of ICA at higher zein and ICA concentrations might be credited to the hydrophobic interactions between the lipophilic compound and the polar groups of zein. Previous studies demonstrated significant lipophilic moieties entrapment within zein nano-sized systems [11,53].

FTIR data explore the optimized ICA–tocozeinolate components’ interaction. FTIR results showed abolishment of stretching aliphatic CH2 before 3000 cm^−1^ that may be attributed to involvement in nonpolar attraction forces and hydroxyl groups’ intermolecular hydrogen bonding between various formula components. Additionally, results indicated a reduction in intensity and broadness of the C=O group that could be due to participation in hydrogen bonding as a hydrogen bond acceptor or because of bulkiness and steric hindrance resulting from the formation of the nanospheres. XRD data showed ICA loading into tocozeinolate nanospheres (optimized formula), revealing the transformation of ICA crystalline peaks into an amorphous form (Figure 7). ICA amorphous form transformation would result in an enhanced dissolution rate and improved bioavailability as a result of the high-energy and disordered state of the amorphous form when compared to the crystalline form [54]. Based on these results, the optimized ICA–tocozeinolate nanosphere formula could represent a promising formula for improving the delivery and efficacy of ICA and other promising therapeutic agents.

## 5. Conclusions

In the current study, a D-optimal response surface experimental design was implemented for the formulation and optimization of ICA–tocozeinolate nanospheres. The design was implemented aiming to minimize nanospheres’ size and maximize zeta potential and entrapment efficiency. The optimized ICA–tocozeinolate nanospheres indicated that treatment with optimized ICA–tocozeinolate nanospheres significantly decreased the average time of ML and EL to almost half and IL time by 41% when compared to raw ICA. The findings from this investigation revealed that the novel optimized ICA–tocozeinolate nanospheres could represent a promising formula for improving the delivery and efficacy of therapeutic agents. 

## 6. Patents

This work is protected under United States Patent and Trademark Office (USPTO) application number: 17/687,754.

## Figures and Tables

**Table 1:** Factors’ levels (coded and actual) and responses’ desirability constraints in the response surface D-optimal design used for optimization of ICA–tocozeinolate nanospheres.

Factors	Levels		
	−1	0	+1
**X_1_:** ICA conc (% *w*/*v*)	0.4	0.6	0.8
**X_2_:** TPGS concentration (% *w*/*v*)	4	5	6
**X_3_:** Zein Concentration (% *w*/*v*)	0.4	0.6	0.8
**X_4_:** SDC concentration (% *w*/*v*)	0.2	0.3	0.4
**Responses**	**Desirability constraints**		
**Y_1_:** Particle size (nm)	Minimize		
**Y_2_:** Zeta potential (mV)	Maximize		
**Y_3_:** Entrapment efficiency %	Maximize		

Abbreviations: ICA, icariin; TPGS, D-α-tocopheryl polyethylene glycol 1000 succinate; SDC, sodium deoxycholate.

**Table 2:** Combination of independent variable levels in ICA–tocozeinolate nanosphere experimental runs and their corresponding responses.

RUN	Independent Variables				Dependent Variables		
	ICA Concentration (X_1_, % *w*/*v*)	TPGS Concentration (X_2_, % *w*/*v*)	Zein Concentration (X_3_, % *w*/*v*)	SDC Concentration (X_4_, % *w*/*v*)	PS ± SD (Y_1_, nm)	ZP ± SD (Y_2_, mV)	EE ± SD (Y_2_, %)
1	0.40	4.00	0.80	0.40	188.2 ± 18.6	−7.36 ± 1.3	61.2 ± 5.2
2	0.80	4.00	0.80	0.40	259.6 ± 20.1	−6.83 ± 0.7	72.1 ± 6.7
3	0.80	4.00	0.80	0.20	421.3 ± 29.3	+0.96 ± 0.1	78.5 ± 4.3
4	0.60	5.00	0.60	0.30	279.2 ± 24.2	−4.13 ± 0.5	60.3 ±7.2
5	0.80	5.33	0.80	0.27	303.1 ± 25.7	−1.95 ± 0.1	69.4 ± 3.5
6	0.80	6.00	0.60	0.20	333.6 ± 27.1	−0.63 ± 0.1	65.7 ± 5.8
7	0.60	4.00	0.40	0.20	538.7 ± 37.4	−0.21 ± 0.03	63.3 ± 3.1
8	0.80	4.00	0.40	0.40	329.8 ± 26.2	−9.91 ± 0.8	63.6 ± 6.9
9	0.80	6.00	0.60	0.20	328.4 ± 29.7	−0.71± 0.2	64.9 ± 8.1
10	0.40	5.00	0.80	0.20	268.1 ± 26.5	+1.94 ± 0.2	62.8 ± 5.2
11	0.60	6.00	0.80	0.20	242.4 ± 19.4	+2.33 ± 0.1	64.1 ± 4.6
12	0.80	4.67	0.40	0.20	494.9 ± 31.6	−1.31 ± 0.1	68.5 ± 5.1
13	0.40	5.00	0.40	0.40	203.1 ± 23.7	−11.63 ± 0.9	36.2 ± 3.1
14	0.40	6.00	0.80	0.30	139.6 ± 16.9	−6.15 ± 0.5	51.7 ± 3.1
15	0.80	4.00	0.40	0.40	334.3 ±21.4	−10.45 ± 0.9	63.9 ± 2.9
16	0.40	6.00	0.60	0.40	114.2 ± 15.4	−8.41 ± 0.6	35.8 ± 2.1
17	0.80	6.00	0.40	0.40	221.4 ± 31.2	−13.27 ± 1.1	57.9 ± 4.4
18	0.80	6.00	0.80	0.40	148.7 ± 23.1	−7.83 ± 0.3	64.4 ± 5.1
19	0.67	6.00	0.40	0.27	311.8 ± 35.2	−4.82 ± 0.3	58.8 ± 6.1
20	0.40	4.00	0.80	0.40	194.9 ± 24.7	−6.97 ± 0.4	60.5 ± 7.1
21	0.80	4.00	0.80	0.20	406.2 ± 54.3	+0.35 ± 0.03	76.9 ± 5.2
22	0.40	6.00	0.40	0.20	291.2 ± 17.5	−2.61 ± 3.6	44.2 ± 3.1
23	0.40	4.00	0.40	0.27	364.3 ± 22.1	−3.44 ± 2.7	55.1 ± 4.8

Abbreviations: ICA, icariin; TPGS, D-α-tocopheryl polyethylene glycol 1000 succinate; SDC, sodium deoxycholate PS, particle size; ZP, zeta potential; EE%, entrapment efficiency.

**Table 3:** Fit statistics of ICA–tocozeinolate nanospheres’ responses according to the best-fitting model.

Responses	Model	Sequential *p*-Value	Lack of Fit *p*-Value	R^2^	Adjusted R^2^	Predicted R^2^	Adequate Precision	PRESS
**Y_1_: PS (nm)**	2FI	0.0036	0.1138	0.9947	0.9903	0.9806	53.77	4845.41
**Y_2_: ZP (mV)**	Linear	<0.0001	0.1582	0.9733	0.9673	0.9563	34.61	17.99
**Y_3_: EE (%)**	Quadratic	0.0030	0.2436	0.9977	0.9937	0.9772	59.88	58.20

Abbreviations: ICA, icariin; PS, particle size; ZP, zeta potential; EE%, entrapment efficiency; 2FI, two-factor interaction; PRESS, predicted residual error sum of squares.

## Data Availability

Data are contained in the article.

## References

[1] Wang L., Li Y., Guo Y., Ma R., Fu M., Niu J., Gao S., Zhang D. (2016). Herba Epimedii: An Ancient Chinese Herbal Medicine in the Prevention and Treatment of Osteoporosis. Curr. Pharm. Des..

[2] Tan H.M., Low W.Y., Ng C.J., Chen K.K., Sugita M., Ishii N., Marumo K., Lee S.W., Fisher W., Sand M. (2007). Prevalence and correlates of erectile dysfunction (ED) and treatment seeking for ED in asian men: The asian men’s attitudes to life events and sexuality (MALES) study. J. Sex. Med..

[3] Angeloni C., Barbalace M.C., Hrelia S. (2019). Icariin and its metabolites as potential protective phytochemicals against Alzheimer’s disease. Front. Pharmacol..

[4] Fang J., Zhang Y. (2017). Icariin, an anti-atherosclerotic drug from chinese medicinal herb horny goat weed. Front. Pharmacol..

[5] Jia G., Zhang Y., Li W., Dai H. (2019). Neuroprotective role of icariin in experimental spinal cord injury via its antioxidant, anti-neuroinflammatory and anti-apoptotic properties. Mol. Med. Rep..

[6] Kong L., Liu J., Wang J., Luo Q., Zhang H., Liu B., Xu F., Pang Q., Liu Y., Dong J. (2015). Icariin inhibits TNF-α/IFN-γ induced inflammatory response via inhibition of the substance P and p38-MAPK signaling pathway in human keratinocytes. Int. Immunopharmacol..

[7] Wu H., Kim M., Han J. (2016). Icariin metabolism by human intestinal microflora. Molecules.

[8] Cao Y.F., He R.R., Cao J., Chen J.X., Huang T., Liu Y. (2012). Drug-drug interactions potential of icariin and its intestinal metabolites via inhibition of intestinal UDP-glucuronosyltransferases. Evid.-Based Complement. Altern. Med..

[9] Deo N., Jockusch S., Turro N.J., Somasundaran P. (2003). Surfactant interactions with zein protein. Langmuir.

[10] Ezzat S.M., Ezzat M.I., Okba M.M., Hassan S.M., Alkorashy A.I., Karar M.M., Ahmed S.H., Mohamed S.O., Fernandes G. (2019). Brain Cortical and Hippocampal Dopamine: A New Mechanistic Approach for Eurycoma longifolia Well-Known Aphrodisiac Activity and Its Chemical Characterization. Evid.-Based Complement. Altern. Med..

[11] Weissmueller N.T., Lu H.D., Hurley A., Prud’Homme R.K. (2016). Nanocarriers from GRAS Zein Proteins to Encapsulate Hydrophobic Actives. Biomacromolecules.

[12] Xu J., Zhang Y. (2020). Traditional Chinese Medicine treatment of COVID-19. Complement. Ther. Clin. Pract..

[13] Wu Y., Wang L., Hu K., Yu C., Zhu Y., Zhang S., Shao A. (2018). Mechanisms and therapeutic targets of depression after intracerebral hemorrhage. Front. Psychiatry.

[14] Zheng J., Hu S., Wang J., Zhang X., Yuan D., Zhang C., Liu C., Wang T., Zhou Z. (2021). Icariin improves brain function decline in aging rats by enhancing neuronal autophagy through the AMPK/mTOR/ULK1 pathway. Pharm. Biol..

[15] Giuliano F., Allard J. (2001). Dopamine and sexual function. Int. J. Impot. Res..

[16] Alhakamy N.A., Fahmy U.A., Badr-Eldin S.M., Ahmed O.A.A., Asfour H.Z., Aldawsari H.M., Algandaby M.M., Eid B.G., Abdel-Naim A.B., Awan Z.A. (2020). Optimized icariin phytosomes exhibit enhanced cytotoxicity and apoptosis-inducing activities in ovarian cancer cells. Pharmaceutics.

[17] Parveen S., Sahoo S.K. (2008). Polymeric nanoparticles for cancer therapy. J. Drug Target..

[18] Regier M.C., Taylor J.D., Borcyk T., Yang Y., Pannier A.K. (2012). Fabrication and characterization of DNA-loaded zein nanospheres. J. Nanobiotechnol..

[19] Ates M., Kaynak M.S., Sahin S. (2016). Effect of permeability enhancers on paracellular permeability of acyclovir. J. Pharm. Pharmacol..

[20] Conacher M., Alexander J., Brewer J.M. (2001). Oral immunisation with peptide and protein antigens by formulation in lipid vesicles incorporating bile salts (bilosomes). Vaccine.

[21] Khurana R.K., Gaspar B.L., Welsby G., Katare O.P., Singh K.K., Singh B. (2018). Improving the biopharmaceutical attributes of mangiferin using vitami E-TPGS co-loaded self-assembled phosholipidic nano-mixed micellar systems. Drug Deliv. Transl. Res..

[22] Ahmed O.A.A., El-Say K.M., Aljaeid B.M.B.M., Badr-Eldin S.M.S.M., Ahmed T.A.T.A., Ahmed O.A.A. (2018). Optimized vinpocetine-loaded vitamin E D-α-tocopherol polyethylene glycol 1000 succinate-alpha lipoic acid micelles as a potential transdermal drug delivery system: In vitro and ex vivo studies. Int. J. Nanomed..

[23] Mi Y., Zhao J., Feng S.-S. (2012). Vitamin E TPGS prodrug micelles for hydrophilic drug delivery with neuroprotective effects. Int. J. Pharm..

[24] Gorain B., Choudhury H., Pandey M., Kesharwani P. (2018). Paclitaxel loaded vitamin E-TPGS nanoparticles for cancer therapy. Mater. Sci. Eng. C.

[25] Neophytou C.M., Constantinou A.I. (2015). Drug delivery innovations for enhancing the anticancer potential of vitamin e isoforms and their derivatives. BioMed Res. Int..

[26] Fahmy U.A., Fahmy O., Alhakamy N.A. (2021). Optimized icariin cubosomes exhibit augmented cytotoxicity against SKOV-3 ovarian cancer cells. Pharmaceutics.

[27] Polat D.C., Coskun M. (2016). Quantitative determination by HPLC-DAD of icariin, epimedin A, epimedin B, and epimedin C in epimedium (berberidaceae) species growing in Turkey. Nat. Prod. Commun..

[28] Malmnäs C.O., Meyerson B.J. (1971). P-chlorophenylalanine and copulatory behaviour in the male rat. Nature.

[29] Fahmy U.A., Badr-Eldin S.M., Ahmed O.A.A., Aldawsari H.M., Tima S., Asfour H.Z., Al-Rabia M.W., Negm A.A., Sultan M.H., Madkhali O.A.A. (2020). Intranasal niosomal in situ gel as a promising approach for enhancing flibanserin bioavailability and brain delivery: In vitro optimization and ex vivo/in vivo evaluation. Pharmaceutics.

[30] Krasnici S., Werner A., Eichhorn M.E., Schmitt-Sody M., Pahernik S.A., Sauer B., Schulze B., Teifel M., Michaelis U., Naujoks K. (2003). Effect of the surface charge of liposomes on their uptake by angiogenic tumor vessels. Int. J. Cancer.

[31] Wang H.X., Zuo Z.Q., Du J.Z., Wang Y.C., Sun R., Cao Z.T., Ye X.D., Wang J.L., Leong K.W., Wang J. (2016). Surface charge critically affects tumor penetration and therapeutic efficacy of cancer nanomedicines. Nano Today.

[32] Gangadhar K.N., Adhikari K., Srichana T. (2014). Synthesis and evaluation of sodium deoxycholate sulfate as a lipid drug carrier to enhance the solubility, stability and safety of an amphotericin B inhalation formulation. Int. J. Pharm..

[33] Tan H.L., Chan K.G., Pusparajah P., Saokaew S., Duangjai A., Lee L.H., Goh B.H. (2016). Anti-cancer properties of the naturally occurring aphrodisiacs: Icariin and its derivatives. Front. Pharmacol..

[34] Jain A.K., Thareja S. (2019). In vitro and in vivo characterization of pharmaceutical nanocarriers used for drug delivery. Artif. Cells Nanomed. Biotechnol..

[35] Gunasekaran T., Haile T., Nigusse T., Dhanaraju M.D. (2014). Nanotechnology: An effective tool for enhancing bioavailability and bioactivity of phytomedicine. Asian Pac. J. Trop. Biomed..

[36] Chen H., Khemtong C., Yang X., Chang X., Gao J. (2011). Nanonization strategies for poorly water-soluble drugs. Drug Discov. Today.

[37] Beloqui A., del Pozo-Rodríguez A., Isla A., Rodríguez-Gascón A., Solinís M.Á. (2017). Nanostructured lipid carriers as oral delivery systems for poorly soluble drugs. J. Drug Deliv. Sci. Technol..

[38] Yadav S.K., Mishra S., Mishra B. (2012). Eudragit-based nanosuspension of poorly water-soluble drug: Formulation and in vitro-in vivo evaluation. AAPS PharmSciTech.

[39] Guo Y., Luo J., Tan S., Otieno B.O., Zhang Z. (2013). The applications of Vitamin e TPGS in drug delivery. Eur. J. Pharm. Sci..

[40] Sadoqi M., Lau-Cam C.A., Wu S.H. (2009). Investigation of the micellar properties of the tocopheryl polyethylene glycol succinate surfactants TPGS 400 and TPGS 1000 by steady state fluorometry. J. Colloid Interface Sci..

[41] Dintaman J.M., Silverman J.A. (1999). Inhibition of P-glycoprotein by D-α-tocopheryl polyethylene glycol 1000 succinate (TPGS). Pharm. Res..

[42] Varma M.V.S., Panchagnula R. (2005). Enhanced oral paclitaxel absorption with vitamin E-TPGS: Effect on solubility and permeability in vitro, in situ and in vivo. Eur. J. Pharm. Sci..

[43] Zou T., Gu L. (2013). TPGS emulsified zein nanoparticles enhanced oral bioavailability of daidzin: In vitro characteristics and in vivo performance. Mol. Pharm..

[44] Sun Y., Zhang M., Liu C., Zhou S., Zhang W., Wang T., Zhou M., Liu X., Wang Y., Sun Y. (2017). Development of Liposome containing sodium deoxycholate to enhance oral bioavailability of itraconazole. Asian J. Pharm. Sci..

[45] Sharma S., Shukla P., Misra A., Mishra P.R. (2014). Interfacial and colloidal properties of emulsified systems: Pharmaceutical and biological perspective. Colloid and Interface Science in Pharmaceutical Research and Development.

[46] Yingchoncharoen P., Kalinowski D.S., Richardson D.R. (2016). Lipid-based drug delivery systems in cancer therapy: What is available and what is yet to come. Pharmacol. Rev..

[47] Barua S., Mitragotri S. (2014). Challenges associated with penetration of nanoparticles across cell and tissue barriers: A review of current status and future prospects. Nano Today.

[48] Zhang Y.R., Lin R., Li H.J., He W.L., Du J.Z., Wang J. (2019). Strategies to improve tumor penetration of nanomedicines through nanoparticle design. Wiley Interdiscip. Rev. Nanomed. Nanobiotechnol..

[49] Badr-Eldin S.M., Aldawsari H.M., Ahmed O.A.A., Alhakamy N.A., Neamatallah T., Okbazghi S.Z., Fahmy U.A. (2021). Optimized semisolid self-nanoemulsifying system based on glyceryl behenate: A potential nanoplatform for enhancing antitumor activity of raloxifene hydrochloride in MCF-7 human breast cancer cells. Int. J. Pharm..

[50] Lazaridou M., Christodoulou E., Nerantzaki M., Kostoglou M., Lambropoulou D.A., Katsarou A., Pantopoulos K., Bikiaris D.N. (2020). Formulation and in-vitro characterization of chitosan-nanoparticles loaded with the iron chelator deferoxamine mesylate (DFO). Pharmaceutics.

[51] Gagliardi A., Bonacci S., Paolino D., Celia C., Procopio A., Fresta M., Cosco D. (2019). Paclitaxel-loaded sodium deoxycholate-stabilized zein nanoparticles: Characterization and in vitro cytotoxicity. Heliyon.

[52] Gagliardi A., Paolino D., Iannone M., Palma E., Fresta M., Cosco D. (2018). Sodium deoxycholate-decorated zein nanoparticles for a stable colloidal drug delivery system. Int. J. Nanomed..

[53] Gagliardi A., Voci S., Salvatici M.C., Fresta M., Cosco D. (2021). Brij-stabilized zein nanoparticles as potential drug carriers. Colloids Surf. B Biointerfaces.

[54] Hashem F.M., Al-Sawahli M.M., Nasr M., Ahmed O.A.A. (2015). Optimized zein nanospheres for improved oral bioavailability of atorvastatin. Int. J. Nanomed..

